# Outcomes of Common General Surgery Patients Discharged Over Weekends at a Tertiary Care Hospital in Taif, Saudi Arabia

**DOI:** 10.7759/cureus.27014

**Published:** 2022-07-19

**Authors:** Abeer I Alsulaimani, Khalid M Alzahrani, Khalid M Al Towairgi, Layla M Alkhaldi, Amani H Alrumaym, Zouhor A Alhossaini, Rami F Algethami

**Affiliations:** 1 Medicine, Taif University, Taif, SAU; 2 General Surgery, Taif University, Taif, SAU; 3 General Surgery, Al Hada Armed Forces Hospital, Taif, SAU

**Keywords:** general surgery, discharge planning, readmission, weekend effect, weekend discharge

## Abstract

Introduction: The admission of patients on weekends in multiple health centers has been associated with poorer outcomes relative to care provided during regular weekday hours. This study aimed to assess and compare the health outcomes of patients discharged on weekends and weekdays after undergoing surgery in a tertiary hospital in Taif, Saudi Arabia.

Materials and methods: The data of patients were collected from hospital records in a retrospective manner, and the outcomes were assessed after discharge. Patients discharged on Friday and Saturday were considered weekend discharges, and those discharged on other days were considered weekday discharges. Data related to readmission and emergency department (ED) visits included the primary diagnosis, number of days post-primary discharge, length of stay, chief complaint, and the number of ED visits. A logistic regression model was done to assess the predictive factor for 30-readmission after surgery.

Results: The frequency of discharge over the weekend was 9.1%. About 6.5% and 7.3% were found to have 30-day readmission and 30-day ED visits, respectively. A statistically significant association was not observed between weekend discharge and the development of postoperative complications (p>0.05). A multinomial logistic regression showed that patients who had emergency admission, postoperative complications, and the presence of cancer were found to be independently associated with 30-day readmission after discharge (P<0.05).

Conclusion: Proactive strategies to reduce costly readmissions after surgery can be designed once the high-risk patient subset is identified.

## Introduction

The admission of patients to several health centers on the weekend has been associated with worse outcomes compared to weekday admissions. This phenomenon is known as the "weekend effect," and it is believed to be caused by a shortage of staffing and resources, resulting in care deficiencies and poor outcomes [1]. Moreover, the weekend effect has been utilized to illustrate the increased risk of death and complications following surgery on weekends compared to weekdays [2]. In large administrative database studies, the 'weekend effect' has also been associated with increased mortality following elective surgery [3]. This effect, however, may not exist in all healthcare systems and may be partially attributable to the increased severity of patients who present to the hospital on weekends, indicating delayed care-seeking [4]. Prior research [5] has demonstrated that the discharge of fewer patients on weekends compared to weekdays leads to unnecessary increases in length of stay.

Elective surgeries are rarely conducted on weekends at many hospitals, and the risk profiles of elective cases chosen for weekend procedures may differ from those chosen for weekday operations [1]. In Canada, patients who have elective surgery on the weekend have a two-fold higher risk of death than those who have surgery on a Monday. In the United Kingdom, patients who have elective surgery on the weekend have an 80% higher risk of death than those who have surgery on a Monday [6]. A study done by Bell et al. reported that patients admitted on weekends with ruptured abdominal aortic aneurysms, acute epiglottitis, and pulmonary embolism are associated with higher in-hospital mortality rates than those who were admitted on weekdays for the same problems [7]. An Australian study done among stroke/transient ischemic attack patients reported that patients discharged on weekends received suboptimal care and had higher long-term mortality compared to those discharged on weekdays [8]. 

In the state of California, United States, it was reported that patients admitted for cancer of the ovary/uterus, duodenal ulcer, and cardiovascular symptoms were associated with higher risk-adjusted mortality than patients with the same problems on weekdays [9]. A nationwide analysis done in the United States among patients with acute variceal hemorrhage and acute nonvariceal who were discharged on weekends had higher adjusted in-hospital mortality compared to those discharged on weekdays [10]. A meta-analysis that examined the presence of a weekend effect on hospital inpatient mortality reported that patients admitted on the weekends had a significantly higher overall mortality compared to those who were admitted on weekdays [11]. Another cohort study done in Canada reported that surgical interventions done on hospitalized patients during weekends were associated with a significant proportional increase in 30-day all-cause mortality compared to those done on weekdays [12].

A weekend impact may also affect patients undergoing urgent or emergent procedures. In this population, however, a weekend effect assessment must take into account the potential influence of inpatient preoperative care quality and delays to urgent or emergent surgery on outcomes [1]. Appendectomies, cholecystectomies, and hernia repairs are the most commonly performed surgical procedures in the general surgery field, and there are fewer studies assessing differences in those patients' outcomes between weekend and weekday discharges. Hence, this study aimed to assess patients' outcomes of common surgical procedures who are admitted and discharged over weekends, and also to identify the predictors of readmission. The objectives of the study include the comparison between patient outcomes of common surgical procedures who are discharged over weekends with weekdays and the identification of key predictors of 30-day readmission who were discharged on the weekend.

## Materials and methods

A retrospective cohort study was conducted on patients who underwent surgical procedures such as appendectomy, cholecystectomy, and hernia repair in a tertiary care hospital in Taif, Saudi Arabia from January 2019 to December 2019. Permission to collect data required for the study was taken from the hospitals' administration after taking ethical approval from the Research and Ethics committee of Armed Forces Hospitals (approval no. 2021-552). All the information related to the patients was kept confidential. A convenience sampling method was applied based on the availability of the patient databases. A pre-tested standardized proforma was used to collect data related to the patients. We exclude those under the age of 18, pregnant women, and other hospitals in Taif due to a lack of a database system.

Details on patient demographics, comorbidities (diabetes mellitus, hypertension, dyslipidemia, coronary artery diseases, and asthma/chronic obstructive pulmonary disease), admission, and follow-up details were all collected from the electronic medical records system of Al-Hada military hospital, Taif. The date and day of admission and discharge, admission type (elective or unplanned), length of stay (LOS), complications, the discharge team, and post-discharge follow-up included all details about admission and discharge. Patients were categorized into two groups according to the day of discharge, namely Group 1: weekend discharge i.e., Friday and Saturday; and Group 2: weekday discharge i.e., Sunday to Thursday. The first unplanned inpatient hospitalization or ED visit for any reason 30 days after discharge from general surgery services was considered hospital readmission, and ED visits. Data related to readmission and ED visits included the primary diagnosis, number of days post-primary discharge, length of stay, chief complaint, and the number of ED visits.

Statistical analysis and data management

All the collected information was tabulated on a Microsoft Excel sheet (Microsoft Corp., Redmond, WA, USA) and then transferred to Statistical Package for Social Sciences (SPSS) version 23 (IBM Corp., Armonk, NY, USA) for data analysis. Categorical data were represented with descriptive statistics in the form of frequencies and percentages using appropriate tables and graphs. Continuous variables were presented using median and interquartile range (IQR) after testing the normality of data distribution. Comparison of continuous variables between categorical variables was tested using the Student's T-test and/or analysis of variance. Any possible association between categorical variables was measured using Pearson's chi-square test. Multivariate logistic regression was used to analyze key predictive factors for 30-day readmission. A p-value <0.05 was considered statistically significant. 

## Results

Our analysis included data from 383 patients discharged from a tertiary hospital in Taif after major surgical procedures. The demographic characteristics showed that 221 (57.1%) were female, and 162 (42.3%) were male. Eighty-eight (23%) belonged to the 46 to 55 year age group, 236 (61.6%) had cholecystectomy, 35 (9.1%) had weekend discharge, 137 (35.8%) had comorbidity and/or risk factors, 118 (30.8%) had unplanned (emergency) admission, 86 (22.5%) had open surgery, 17 (4.4%) had complication during admission, 31 (8.1%) had postoperative complications, 25 (6.5%) made 30-day re-admission, 28 (7.3%) made 30-day emergency department visits, and 342 (89.3%) had 30-day follow-up (Table 1). 

**Table 1:** Baseline characteristics of the patients (n=383)

Variables		Frequency	Percentage
Gender	Female	221	57.7
	Male	162	42.3
Age (years)	<25	57	14.9
	26-35	57	14.9
	36-45	74	19.3
	46-55	88	23.0
	56-65	51	13.3
	>65	56	14.6
Surgical procedure	Appendectomy	49	12.8
	Cholecystectomy	236	61.6
	Hernia repair	98	25.6
Discharge day	Weekday	348	90.9
	Weekend	35	9.1
Comorbidity and risk factors	Absent	246	64.2
	Present	137	35.8
Admission type	Elective	265	69.2
	Unplanned (emergency)	118	30.8
Type of surgery	Laparoscopic	297	77.5
	Open	86	22.5
Complication during admission	No	366	95.6
	Yes	17	4.4
Having postoperative complications	No	352	91.9
	Yes	31	8.1
30-day re-admission	No	358	93.5
	Yes	25	6.5
30- day emergency department visits	No	355	92.7
	Yes	28	7.3
30-day follow-up	No	41	10.7
	Yes	342	89.3

**Figure 1:**
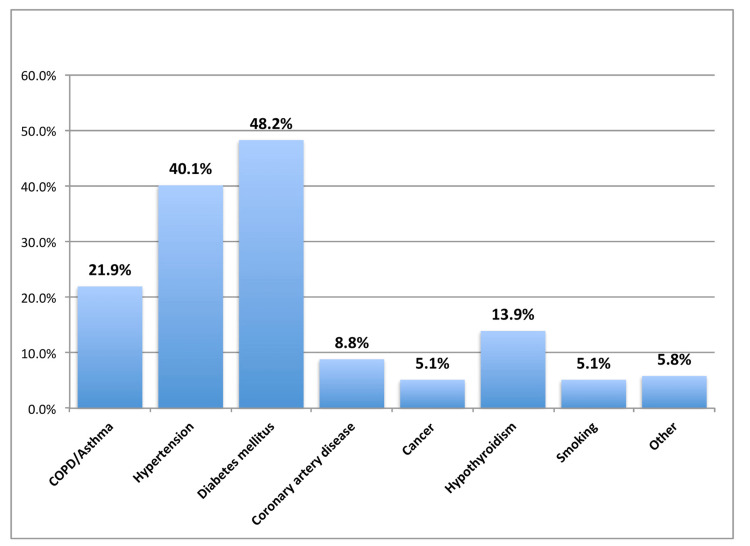
Comorbidities and risk factors (n=137) COPD: Chronic obstructive pulmonary disease

Among those who had reported some comorbidity and risk factors, the most commonly reported comorbidity was diabetes mellitus (48.2%), followed by hypertension (40.1%), chronic obstructive pulmonary disease (COPD)/asthma (21.9%), hypothyroidism (13.9%), coronary artery disease (8.8%), and smoking (5.1%) (Figure 1).

The comparison of baseline characteristics of 383 patients discharged on the weekend and weekdays is given in Table 2. There were no statistically significant differences observed for gender (p=0.944), comorbidity (p=0.351), type of surgery (p=0.363), complications during admission (p=0.634), postoperative complications (p=0.448), 30-day readmission (p=0.218), 30-day emergency department visits (p=0.326), 30-day follow-up (p=0.472), and length of hospital stay (p=0.479). However, compared with patients discharged on weekdays, patients discharged on weekends had lesser age (median 46.5 vs 36 years, respectively; p=0.017). Also, patients discharged over the weekend had undergone unplanned or emergency (18.6%) admission compared to elective surgery (4.9%), p<0.001 (Table 2).

**Table 2:** Comparison of baseline characteristics of 383 patients discharged on the weekend and weekdays IQR: Interquartile range

Variables		Discharge type		p-value
		Weekday	Weekend	
Sex	Male	147 (90.7%)	15 (9.3%)	0.944
	Female	201 (91.0%)	20 (9.0%)	
Age (median, IQR)		46.5 (25, 84	36 (25, 90)	<0.017
Comorbidity	Absent	221 (89.8%)	25 (10.2%)	0.351
	Present	127 (92.7%)	10 (7.3%)	
Admission type	Elective	252 (95.1%)	13 (4.9%)	<0.001
	Unplanned (emergency)	96 (81.4%)	22 (18.6%)	
Type of surgery	Laparoscopic	272 (91.6%)	25 (8.4%)	0.363
	Open	76 (88.4%)	10 (11.6%)	
Complications during admission	No	332 (90.7%)	34 (9.3%)	0.634
	Yes	16 (94.1%)	1 (5.9%)	
Postoperative complications	No	321 (91.2%)	31 8.8%	0.448
	Yes	27 (87.1%)	4 (12.9%)	
30-day readmission	No	327 (91.3%)	31 (8.7%)	0.218
	Yes	21 (84.0%)	4 (16.0%)	
30-day emergency department visits	No	324 (91.3%)	31 (8.7%)	0.326
	Yes	24 (85.7%)	4 (14.3%)	
30-day follow-up	No	36 (87.8%)	5 (12.2%)	0.472
	Yes	312 (91.2%)	30 (8.8%)	
Length of hospital stay (median, IQR)		2 (2,31)	3 (2,12)	0.479

**Figure 2:**
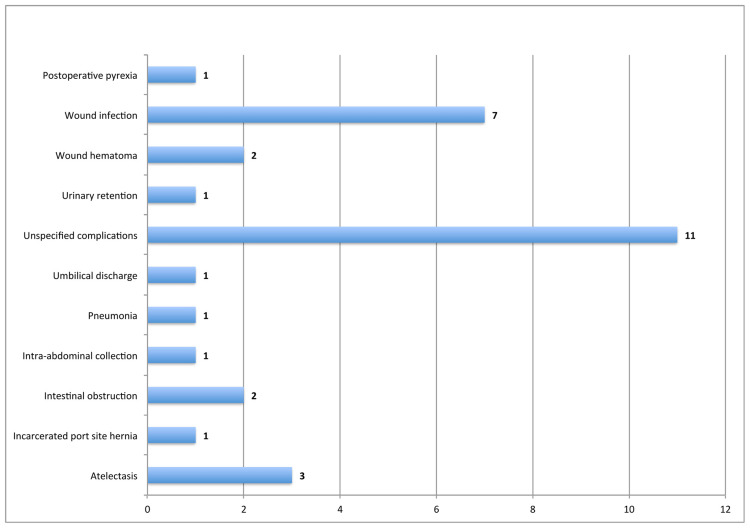
Frequency of postoperative complications (n=31)

It was observed that about 31 (8.1%) patients had developed postoperative complications, out of which 11 patients had unspecified complications, and seven had wound infections at the site of surgery (Figure 2).

A multinomial logistic regression model analysis showed that patients who had emergency admission (odds ratio (OR)=3.53 (1.45-8.59), p=0.006), postoperative complications (OR=44.45 (5.23-377.28), p=0.001) and cancer (OR=13.56 (1.19-154.9), p=0.035) were found to be independently associated with 30-day readmission (Table 3).

**Table 3:** Multinomial logistic regression for 30-day readmission OR: Odds ratio, CI: Confidence interval, COPD: Chronic obstructive pulmonary disease

Dependent variables	Odds ratio (OR)	95% CI for OR	p-value
Age	1.16	0.72 - 1.85	0.533
Length of hospital stay	0.97	0.87-1.09	0.590
Weekend discharge	1.37	0.16-11.70	0.770
Comorbidity	1.08	0.43 -2.67	0.934
Surgery procedure	0.92	0.27- 3.14	0.903
Type of surgery	1.04	0.18 – 6.02	0.960
Admission type = Emergency	3.53	1.45 -8.59	0.006
Complications during admission	0.28	0.16 -2.857	0.384
Postoperative complications	44.45	5.23 – 377.28	0.001
30-day emergency department visits	1.20	1.06 -1.34	0.940
30-day follow-up	1.35	0.10- 17.47	0.818
Hypertension	0.70	0.64-6.94	0.760
Diabetes mellitus	5.24	1.21-6.12	0.995
COPD	0.73	0.04-8.45	0.804
Coronary artery disease	0.94	0.22-40.09	0.974
Cancer	13.56	1.19-154.90	0.035
Hypothyroidism	0.433	0.02-9.92	0.600
Smoking	1.10	0.09-9.92	0.972
Length of stay	0.930	0.78-1.10	0.421

## Discussion

We utilized hospital records and follow-up records of the patients who underwent surgical procedures such as appendectomy, cholecystectomy, and hernia repair. We hypothesized that weekend hospital discharge would be associated with poor or worse outcomes than weekday discharges. However, our study findings did not support the above study hypothesis. The only factor that was independently associated with the weekend discharge was unplanned (emergency) admission for the surgical procedure. Despite apparent similarities to prior studies, which found that weekend discharge for patients undergoing major surgery was not related to higher readmission rates or visits to the emergency departments [13-15], the current study question was set particularly to examine the weekend effect of hospital discharge. Morbidity and mortality, intensive care unit (ICU) and ED readmissions, and delays in appropriate diagnostic imaging and management are just some of the worse outcomes that can be affected by the weekend impact while patients are being treated over the weekend [15]. A study done by Bell et al. on patients with ruptured abdominal aortic aneurysms, pulmonary emboli, or acute epiglottitis who were admitted through the EDs on the weekend, demonstrated increased mortality compared to those admitted on the weekdays [7].

The current study findings showed that 30-day readmission and 30-day ED visits were comparatively more in open surgical procedures than laparoscopic ones, even though no statistically significant differences were observed. The 30-day hospital readmission with appendectomy, cholecystectomy and hernia repair was found to be 8.2%, 5.5%, and 8.2%, respectively. Similar rates for 30-day readmission for patients with the same surgical procedures have been reported by previous studies [16-19]. Immediately following discharge from the hospital, patient follow-up with surgeons is vital to guarantee successful rehabilitation. Our findings showed that about 342 (89.3%) had a follow-up 30 days after surgery, which did not show any significant association with the type of discharge. After a discharge, many factors contribute to a lower rate of follow-up visits. Fidel et al. reported that younger age, surgery during the index hospital, and longer hospital stays were significantly associated with lesser follow-ups [20]. Patients may not be aware of the significance of a follow-up appointment, and physicians may not remember to schedule one. Physicians who discharge patients on weekends should encourage early follow-up in order to improve treatment continuity and improved outcomes.

The "weekend effect" can be attributed to various factors, including the fact that many patients with long-term medical needs are discharged from the hospital on weekends. Insufficient access to diagnostic or therapeutic interventions, unavailability of skilled ancillary staff, or unavailability of post-discharge service scheduling are just some of the reasons that can cause physicians to delay discharge until the weekdays [21]. All of these factors may be a result of deficiencies in the hospital setting over the weekends, which cause patients and their families to spend more time in the hospital and endure more significant stress. In order to provide our surgical patients with the highest level of care, physicians must discharge them and ensure they have access to adequate services upon their return home. Increased morbidity (and hence an increased risk of readmission) is possible with weekend discharge due to the higher patient-physician ratio and patient-ancillary staff ratio [22,23]. In order to reduce the impact of the weekend on surgical patients, greater nurse-to-bed ratios, as well as home-care health services, should be implemented. Reductions in readmissions for patients who require home care services may be possible with improved weekend discharge infrastructure. Thus there is also a need to evaluate the cost-effectiveness of weekend ancillary services in the medical and surgical units. We recommend that every patient be discharged by his primary team and that physicians who discharge patients should encourage early follow-up in order to prevent poor outcomes postoperatively.

One of the study's limitations is the inadequacy of the hospital records to include non-index hospital readmissions. Our 30-day readmission rate after three surgical procedures is similar to other published findings, despite the fact that up to one in four readmissions may be at non-index hospitals [24]. Because of the limitations of our database, we did not include readmissions beyond 30 days, chronic steroid use, or hospital volume in our model. In addition, our model is constrained by the fact that it only accounts for a small number of variables, and this may not have controlled for all of the potential confounding factors. The study's retrospective approach is also another limitation.

## Conclusions

The study findings showed that the proportion of patients discharged during a weekend was lower, even though it was not independently associated with 30-day readmission. Emergency admission, patients with postoperative complications, and patients with some forms of cancer were found to be independently associated with increased 30-day readmission. Identifying this subset of high-risk patients will make it possible to develop proactive interventions to decrease the incidence of expensive readmissions. It may be possible to save healthcare expenditures by coordinating safe weekend discharges from the hospital after major surgical procedures.
